# The Potential of Large Language Models in Gynecologic Oncology Telephone Triage

**DOI:** 10.3390/diagnostics16213668

**Published:** 2026-11-06

**Authors:** Iason Psilopatis, Julius Emons, Matteo Pavone, Tibor A. Zwimpfer

**Affiliations:** 1 Department of Gynecology and Obstetrics, University Hospital Basel, University of Basel, 4031 Basel, Switzerland; 2 Department of Gynecology and Obstetrics, Universitätsklinikum Erlangen, Comprehensive Cancer Center Erlangen-EMN (CCC ER-EMN), Friedrich-Alexander-Universität Erlangen-Nürnberg (FAU), 91054 Erlangen, Germany; 3 Dipartimento di Scienze per la salute della Donna e del Bambino e di Sanità Pubblica, UOC Ginecologia Oncologica, Fondazione Policlinico Universitario A. Gemelli, IRCCS, Largo Agostino Gemelli 8, 00168 Rome, Italy; 4 Department of Biomedicine, University Hospital and University of Basel, 4031 Basel, Switzerland

**Keywords:** large language model, gynecologic oncology, triage, consultation

## Abstract

**Introduction:** Nurse-led telephone triage, frequently guided by the United Kingdom Oncology Nursing Society (UKONS) tool, is a cornerstone of supportive oncology care, yet the appropriateness of triage decisions varies across settings. Large Language Models (LLMs) show promise in medical communication but remain unvalidated for autonomous clinical decision-making, particularly in gynecologic oncology, where remote and telephone-based care pose distinct challenges. **Methods and Materials:** Twenty fictitious case vignettes representing common treatment-related toxicities in gynecologic oncology were developed. Three board-certified gynecologic consultants independently assigned UKONS Red–Amber–Green (RAG) triage categories, reaching unanimous consensus that served as the reference standard. Vignettes were submitted verbatim, as single-turn queries with no follow-up prompting, to three publicly available LLMs (OpenEvidence, ChatGPT, Google Gemini). LLM responses were mapped to RAG categories and compared descriptively against the reference standard, stratified by treatment type and platform. **Results:** All three LLMs achieved 100% concordance with the reference standard for all Red cases across both treatment groups, with no high-acuity case downgraded to self-care. Discrepancies clustered at lower-acuity boundaries. In the chemotherapy-alone group, errors were limited to omission of an explicit 24-h follow-up window for 1 Amber-rated complaints. In the combined group, all LLMs over-triaged intermediate-risk complaints toward immediate or same-day evaluation; low-risk complaints showed more variable over-triage. **Conclusions:** LLMs identified unambiguous emergent gynecologic oncology symptoms, preserving the UKONS safety floor, but showed relatively reduced precision and a conservative over-triage bias at lower-acuity margins, especially in the combined-therapy collective. These findings support a supervised, adjunctive role for LLMs, rather than autonomous deployment, pending prospective validation.

## 1. Introduction

Telephone triage has become a cornerstone of supportive oncology care, functioning as a safety net between scheduled clinical encounters for patients experiencing treatment-related toxicity or disease-related symptoms [1]. Nurse-led triage lines, most notably those guided by structured frameworks such as the United Kingdom Oncology Nursing Society (UKONS) tool, have been associated with improved symptom control, enhanced continuity of care, and meaningful reductions in unnecessary emergency department presentations and hospital admissions [2]. This is particularly consequential in oncology, where immunocompromised patients face elevated risks from emergency department exposure and where national systems, such as the UK’s chemotherapy specification, mandate 24-h access to specialist advice for patients undergoing systemic anticancer therapy [3]. Despite these benefits, telephone triage services remain heterogeneous in structure, staffing, and digital integration, and appropriateness of nurse-delivered triage decisions has been reported to range widely, underscoring persistent variability in quality and reliability across settings [4].

In parallel, Large Language Models (LLMs) have rapidly emerged as tools with the potential to reshape both patient-facing and physician-facing communication in medicine. Models such as GPT and Med-PaLM have demonstrated performance approaching that of expert clinicians on medical licensing and specialty examinations, and in direct comparisons, LLM-generated responses to patient questions have been rated by physician judges as higher in quality and empathy than responses written by physicians themselves [5,6]. Beyond question-answering, LLMs are increasingly explored for chatbot-based patient triage, navigation, and follow-up, as well as for assisting physicians with management reasoning and clinical decision support, with early randomized data suggesting LLM assistance can improve physician performance on structured reasoning tasks [7,8]. At the same time, the literature consistently emphasizes important limitations: LLMs can produce inaccurate or incorrect responses to real-world patient queries and have not been validated for autonomous deployment in clinical decision-making or patient communication [9].

Gynecologic oncology poses distinctive challenges for remote and telephone-based care, given the sensitive nature of the anatomy and diagnoses involved, the frequent need for physical examination, and documented patient hesitancy toward telemedicine for initial oncologic consultations despite general openness to its use for follow-up and symptom management [10]. Existing telemedicine research in this population has focused largely on video and telephone consultation satisfaction and feasibility, with comparatively little attention paid to whether emerging conversational artificial intelligence tools could support or augment the triage process itself [11,12]. Given the demonstrated strengths and well-documented limitations of LLMs in general medical consultation, and the established but variably implemented role of nurse-led telephone triage in oncology, this study aims to evaluate the potential application of LLMs in the specific context of gynecologic oncology telephone triage, examining their capacity to support timely and accurate symptom assessment while identifying the safety considerations that must guide their integration into existing triage workflows.

## 2. Materials and Methods

### 2.1. Study Design and Case Vignette Development

Twenty fictitious case vignettes were constructed by a gynecology resident to reflect the spectrum of adverse events commonly reported by patients with gynecologic malignancies contacting an oncology telephone triage service. Ten vignettes portrayed patients receiving chemotherapy alone, and ten portrayed patients receiving combined therapy (immunotherapy and/or targeted therapy, with or without concurrent chemotherapy). Vignettes were distributed across the principal gynecologic cancer types (e.g., ovarian, endometrial, cervical) to capture a broad range of treatment-related toxicities, including hematologic, gastrointestinal, dermatologic, infectious, and immune-related adverse events, as well as symptoms requiring urgent escalation.

The cases were not derived from actual patient encounters, medical records, or telephone call recordings. Instead, they were constructed de novo based on the UKONS telephone triage tool. The relevant symptom headings and descriptions in the UKONS tool were used to structure the clinical presentations, with the wording and format of the vignettes adapted to reflect typical symptom-related telephone consultations in gynecologic oncology. No pilot testing or preliminary validation of the vignettes was performed before the study.

### 2.2. Reference Standard Triage

Each vignette was independently reviewed by three board-certified gynecologic consultants, who assigned a triage category according to the UKONS telephone triage tool. The UKONS tool applies a Red–Amber–Green (RAG) rating to presenting complaints, with Red denoting the most severe presentations requiring urgent review, Amber denoting intermediate-urgency complaints, and Green denoting complaints manageable with routine advice; some complaints are assigned an automatic Red rating, while others are graded according to defined severity thresholds [13].

The decision to use physician consultants as the reference panel was based on their specialist expertise in gynecologic oncology, including the assessment and management of treatment-related toxicities and potentially life-threatening complications associated with systemic anticancer therapies. Their clinical experience was considered particularly relevant for evaluating the medical urgency of the presented scenarios across different treatment regimens, including chemotherapy, immunotherapy, and targeted therapies. Although the UKONS tool was originally developed to support structured nurse-led oncology telephone triage, its standardized RAG classification system provides explicit criteria for categorizing symptom severity and determining the urgency of clinical assessment. Accordingly, the consultants applied the UKONS criteria consistently to establish a standardized expert reference for comparison with the LLM-generated recommendations. The intention was not to replace or replicate the operational role of oncology triage nurses, but to assess the concordance of LLM recommendations with specialist clinical interpretation of the UKONS framework. The three experts reached unanimous agreement on the RAG category for all twenty vignettes, with no discrepancies requiring adjudication; this consensus rating served as the reference standard for subsequent comparison.

### 2.3. LLM Querying

The twenty vignettes were subsequently submitted in the English language, verbatim and with identical accompanying instructions, to three publicly available LLM platforms: OpenEvidence, ChatGPT 5.6 Luna, and Google Gemini Flash 3.6. Each prompt followed the exact same structure:*Case Presentation. How do you consult the patient? Can this patient stay at home?*. Each vignette was entered as the first and only prompt in a newly initiated chat session to prevent carryover effects from prior conversation context. No additional prompting, follow-up questioning, or iterative refinement was performed; the initial response generated by each platform was recorded verbatim for analysis.

All evaluations were conducted on 29 September 2026, at varying times throughout the day, using the standard web interfaces of the LLMs. OpenEvidence was evaluated through its standard web interface, using a private free-tier account and the default platform settings. Unlike ChatGPT and Gemini, OpenEvidence did not display a specific underlying model name or model identifier in the user interface at the time of testing. Therefore, the platform is reported as OpenEvidence rather than being attributed to a specific underlying language model. Its integrated medical literature retrieval and citation functionality remained available during testing, as these features are intrinsic to the platform. No customized settings or additional tools were deliberately activated. In the cases of ChatGPT and Gemini, the model/version labels displayed by the respective interfaces and documented at the time of testing were GPT-5.6 Luna and Gemini Flash 3.6, respectively. Both platforms were accessed through private free-tier accounts. No API access, paid subscriptions, third-party integrations, or customized system configurations were used. The default interface settings were retained throughout testing. No additional web-search functions or manually activated reasoning modes were used. Model identification was based on the version information displayed by the platforms’ user interfaces. As the evaluations were conducted through consumer-facing services rather than fixed API model endpoints, the underlying model identifier for each individual response could not be independently verified through provider-side logs.

### 2.4. Outcome Assessment

Each LLM-generated response was independently mapped to a RAG triage category (Red, Amber, or Green) by the experts using the same UKONS-based criteria. The three consultant experts evaluated the LLM-generated responses without being provided with the stated identity of the platform that generated each response. However, no formal assessment was performed to determine whether the platform could be inferred from the wording, structure, or style of the responses. Responses recommending urgent clinical assessment on the same day were categorized as Red. Responses explicitly recommending a planned clinical review within 24–48 h, without recommending urgent (onsite) same-day assessment, were categorized as 1 Amber. Responses recommending home management without explicitly specifying a planned follow-up appointment were categorized as Green. Where recommendations included alternative timeframes, such as “same-day/next-day,” the experts considered whether the response explicitly included an urgent same-day assessment recommendation when assigning the category. The three experts reached unanimous agreement on the RAG category for all twenty vignettes, with no discrepancies requiring adjudication. The LLM-assigned category for each vignette was then compared against the corresponding unanimous reference standard category.

### 2.5. Statistical Analysis

Given the small study sample, descriptive statistics were used to summarize concordance between each LLM’s triage classification and the reference standard, including overall agreement rates and the distribution of over- and under-triage events, stratified by treatment type (chemotherapy alone versus combined therapy) and by LLM platform.

## 3. Results

A total of 20 fictitious clinical case vignettes were constructed to represent the spectrum of treatment-related toxicities and adverse events encountered in gynecologic oncology telephone triage. The vignettes comprised patients diagnosed with ovarian cancer (n = 8), cervical cancer (n = 7), endometrial cancer (n = 4), and uterine sarcoma (n = 1). Ten vignettes depicted patients receiving chemotherapy alone (e.g., carboplatin, paclitaxel, gemcitabine, cisplatin, topotecan, docetaxel, etoposide), while ten depicted patients on (combined) regimens incorporating immunotherapy, targeted agents, or anti-angiogenics (e.g., pembrolizumab, dostarlimab, bevacizumab, lenvatinib, olaparib). Clinical presentations encompassed a wide variety of toxicities, including gastrointestinal complaints (nausea, vomiting, severe mucositis, constipation, and bloody diarrhea), hematologic and hemorrhagic manifestations (epistaxis with bruising, gross hematuria), febrile and renal emergencies (neutropenic fever, anuria, dysuria), dermatologic reactions (severe rash with pain), and systemic or neurological symptoms (acute chest pain, dyspnea, disorientation, visual disturbances, paresthesia, and fatigue). Table 1 and Table 2 summarize the case vignettes.

According to the UKONS telephone triage reference standard established by expert consensus, 12 cases were classified as Red (requiring urgent same-day or immediate face-to-face evaluation; including one case originally graded 2 Amber that required escalation to urgent review), 5 cases were classified as 1 Amber (requiring review within 24 h), and 3 cases were classified as Green (suitable for self-care advice and routine home management).

**Figure 1:**
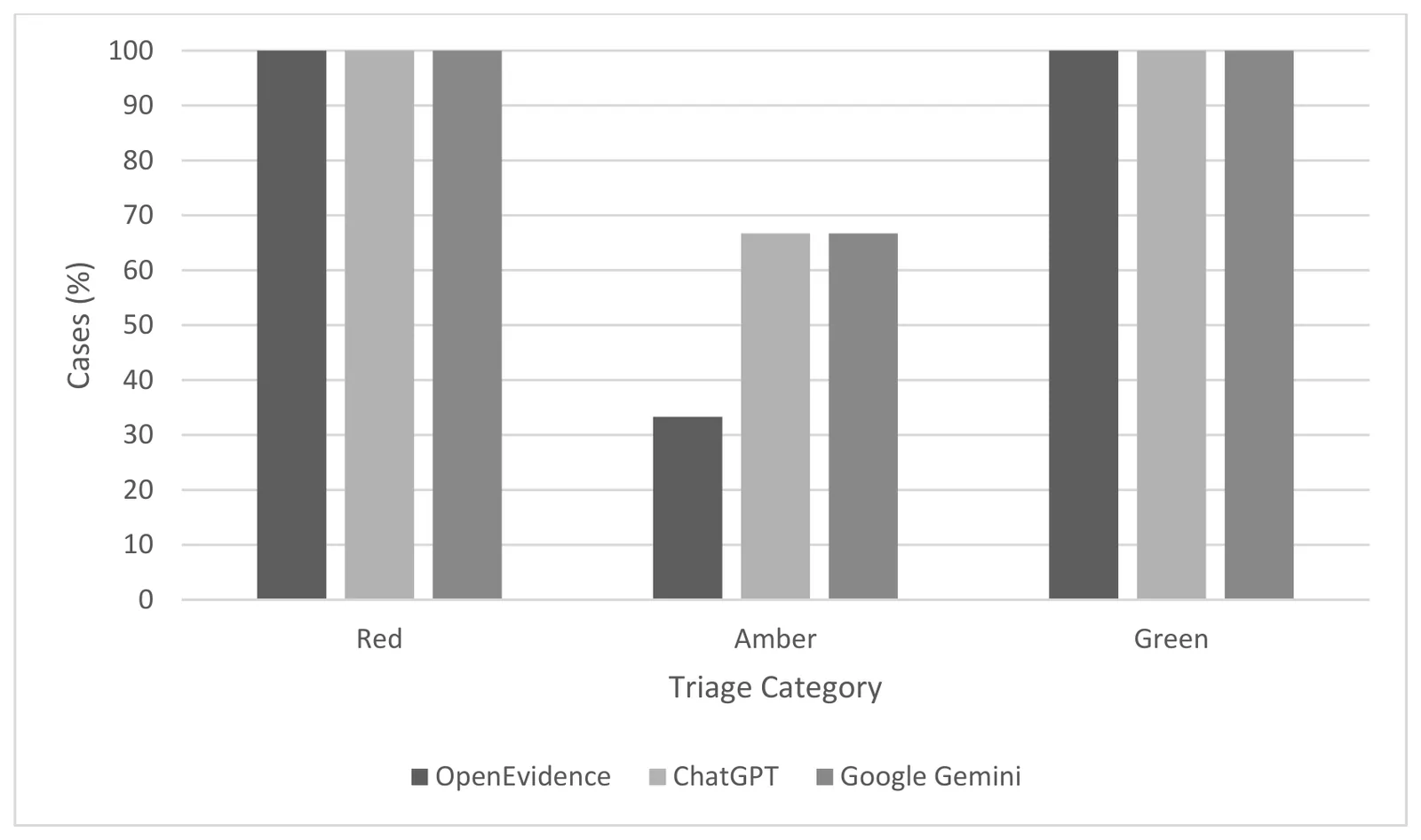
Correct Telephone Triage Category Attribution by the LLMs—Chemotherapy Cases.

In the chemotherapy-alone group (n = 10), the reference standard categorized 6 cases as Red (Cases 2, 3, 4, 5, 8, and 10), 3 cases as 1 Amber (Cases 1, 6, and 7), and 1 case as Green (Case 9). All three LLMs demonstrated complete concordance (100%) with the expert reference standard across the Red and the Green cases. For all 6 Red cases presenting with acute red-flag symptoms—including spontaneous bleeding with ecchymosis (Case 2), febrile dysuria (Case 3), severe stomatitis preventing oral intake (Case 4), rapidly progressive painful rash (Case 5), high fever (Case 8), and acute anuria (Case 10)—each LLM correctly recommended immediate or same-day in-person clinical assessment. For the Green case describing mild, rest-relieved fatigue (Case 9), all models correctly advised supportive home management with explicit return precautions. As for the 1 Amber cases involving mild emesis with preserved intake (Case 1), isolated uncomplicated diarrhea (Case 6), and mild peripheral paresthesia (Case 7), all models appropriately advised initial home management accompanied by structured safety-netting and scheduled clinical re-evaluation within 24 h in Case 1. Nonetheless, only ChatGPT and Google Gemini suggested a scheduled follow-up within 24(–48) h in Case 7, whereas no LLM suggested a scheduled follow-up within 24 h in Case 6. Table 3 and Figure 1 summarize the study results for the chemotherapy-alone group.

**Figure 2:**
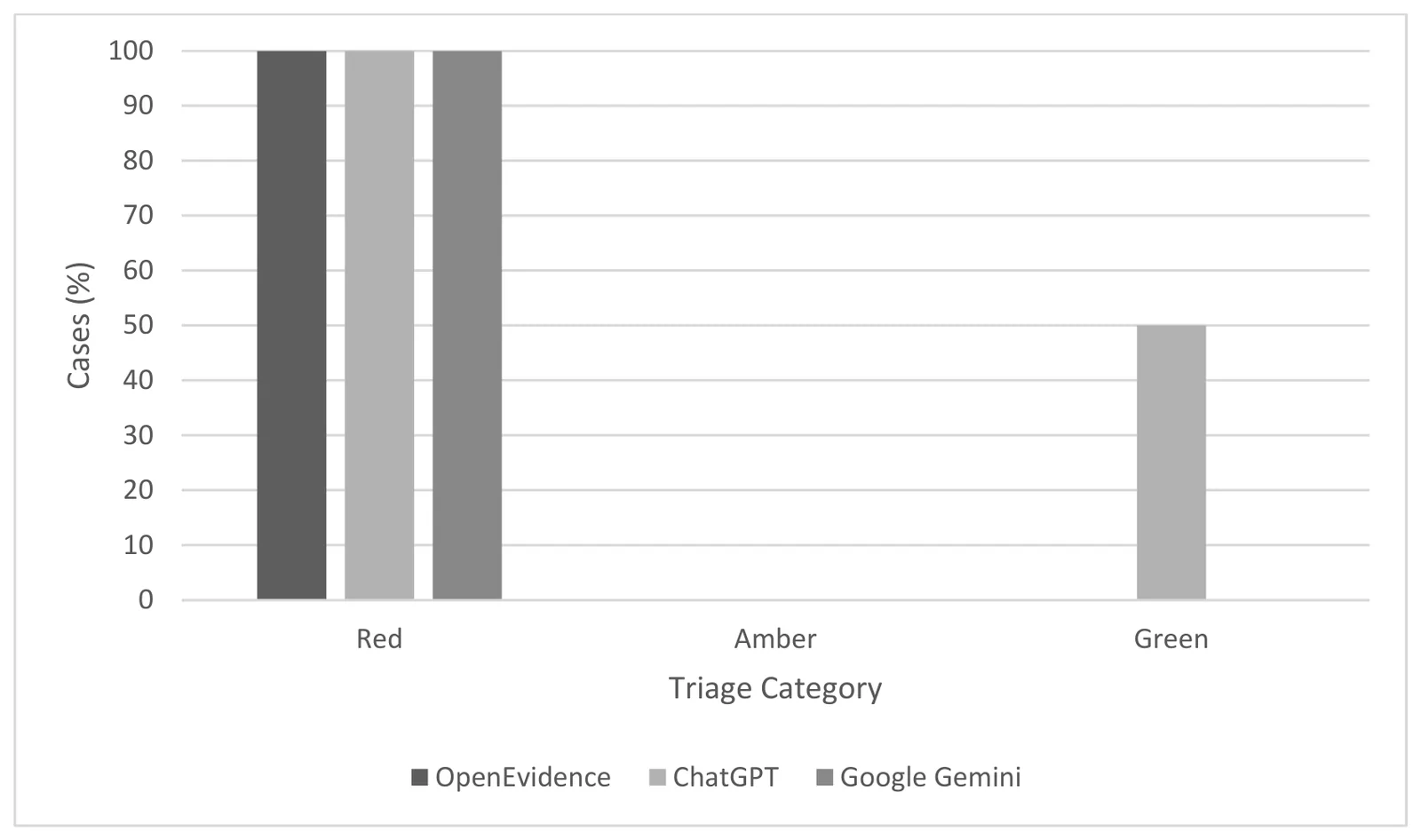
Correct Telephone Triage Category Attribution by the LLMs—Combined Therapy Cases.

In the combination group (n = 10), the reference standard assigned 6 Red cases (Cases 1, 3, 5, 6, 9 [rated 2 Amber/escalated to Red], and 10), two 1 Amber cases (Cases 4 and 8), and 2 Green cases (Cases 2 and 7). All three LLMs correctly identified all 6 Red cases and recommended urgent, same-day, or immediate emergency evaluation, including acute chest pain (Case 1), hematuria with incontinence (Case 3), acute visual disturbance (Case 5), severe bloody diarrhea (Case 6), dyspnea with disorientation (Case 9), and profound weight loss with altered oral intake (Case 10). Discrepancies between the LLMs emerged exclusively in lower-risk and intermediate-risk scenarios (Green and Amber). For the Green cases, all three models recommended re-evaluation within 24–48 h for Case 2 (uncomplicated constipation), whereas in Case 7 (single vomiting episode) OpenEvidence recommended follow-up within 24–48 h, while Google Gemini over-triaged Case 7 by recommending urgent in-person evaluation. For the 1 Amber cases, all three models over-triaged both Case 4 (persistent cough and dyspnea with moderate exertion) and Case 8 (increasing fatigue preventing work) by advising immediate or same-day in-person evaluation instead of 24-h review. Table 4 and Figure 2 summarize the study results for the combined therapy group.

Comparing performance across the two treatment groups, safety was preserved across all LLMs, as no high-risk case (Red or escalated 2 Amber) was downgraded to home self-care without urgent evaluation. All three LLMs achieved 100% accuracy and concordance in identifying all Red cases regardless of treatment or symptom complexity. In the chemotherapy-alone group, all three LLMs classified Case 6 below the reference-standard Amber 1 category by failing to explicitly recommend a planned follow-up within 24 h. The same occurred for Case 7 with OpenEvidence. According to the predefined outcome-assessment criteria, these responses were classified as Green, representing four instances of Amber 1-to-Green sub-triage across the evaluated model-case combinations. Conversely, in the combination therapy group, discrepancies were characterized by over-triage of non-emergent complaints, with all three models over-escalating both 1 Amber cases (Cases 4 and 8) to immediate or same-day in-person evaluation. Analogously, all three LLMs over-escalated the Green Case 2 to review within 24–48 h, while for the Green Case 7, OpenEvidence suggested follow-up in 24–48 h and Google Gemini suggested urgent in-person evaluation. Overall LLM accuracy was 70% for OpenEvidence, 80% for ChatGPT, and 75% for Google Gemini. OpenEvidence showed an over-triage rate of 20% and an under-triage rate of 10%. ChatGPT showed an over-triage rate of 15% and an under-triage rate of 5%. Google Gemini showed an over-triage rate of 20% and an under-triage rate of 5%. Table 5, Table 6 and Table 7 represent 3 × 3 confusion matrices for each model.

## 4. Discussion

This feasibility study found that three publicly available LLMs achieved complete concordance with a (gynecologic) oncology reference standard in identifying every Red (urgent) case across both the chemotherapy-alone and combined-therapy vignettes, with no high-risk case ever downgraded to self-care. Discrepancies clustered exclusively at the lower-acuity margins. In the chemotherapy-alone group, errors were limited to omission of an explicit 24-h follow-up window for 1 Amber-rated complaints (isolated diarrhea, mild paresthesia), rather than any misclassification of urgency itself. In the combination therapy group, by contrast, the three models tended to over-triage genuinely non-emergent or intermediate-risk complaints (persistent cough and dyspnea with moderate exertion, increasing fatigue preventing work, a single vomiting episode) to immediate or same-day evaluation rather than the UKONS-concordant 24-h review. Taken together, the safety floor of the UKONS red-flag framework was preserved across all three LLMs, but precision at the 1 Amber/Green boundary was more variable.

An important aspect of the present study was the prompting strategy. The models were not explicitly instructed to use the UKONS framework or to assign a RAG category. Rather, they were presented with clinical scenarios and asked whether patients could safely remain at home. This approach was intentional, as we aimed to investigate whether general-purpose LLMs could independently generate clinical disposition recommendations consistent with an established UKONS-based expert reference standard, without being guided by the framework itself. In this context, the UKONS classification served as the benchmark for evaluating the models’ recommendations rather than as an instruction provided to the models. A complementary approach, explicitly introducing the UKONS criteria and requesting a RAG classification, would address a different but relevant question: whether LLMs can accurately apply a predefined triage framework when prompted to do so. The framing of the prompt is an important consideration when interpreting these findings. The question, “How do you consult the patient? Can this patient stay at home?”, was directed toward a clinician and asked the model to recommend a clinical disposition based on a predefined vignette. It did not simulate a patient-initiated telephone call or assess the ability of a model to conduct a dynamic triage interaction, obtain additional information, or ask targeted follow-up questions. Accordingly, the findings relate to clinician-facing decision support rather than the performance of LLMs as autonomous patient-facing telephone triage systems. Furthermore, the evaluated platforms differ in their intended use and functionality. OpenEvidence is primarily a clinician-facing medical information and decision-support tool, and its integrated literature retrieval and citation functionality remained available during testing. These differences may have influenced the responses and should be considered when comparing platforms.

This pattern of near-perfect emergent-case identification contrasts with the more variable LLM triage performance described in the broader literature. In a retrospective evaluation of nearly 40,000 real emergency department encounters, agreement between seven contemporary LLMs and physician-assigned Emergency Severity Index levels was only moderate, with documented tendencies toward both over- and under-triage [14]. A systematic review and meta-analysis of LLM diagnostic accuracy for emergency department triage similarly found high specificity but limited sensitivity for identifying the highest-acuity category, such that a substantial proportion of the most urgent cases could be missed [15]. The absence of any under-triage of a Red case in the present study likely reflects differences in vignette design rather than genuine superiority of LLM clinical reasoning: the Red cases constructed here (e.g., neutropenic fever, acute anuria, spontaneous bleeding with ecchymosis, acute chest pain, dyspnea with disorientation) contained unambiguous, textbook red-flag symptoms, whereas real-world encounters frequently present with atypical, overlapping, or incompletely described symptoms, consistent with prior work showing LLM triage accuracy is sensitive to vignette complexity and length [16]. The asymmetric over-triage seen specifically in the combined therapy group is also clinically coherent: symptoms such as cough, dyspnea, and fatigue can herald pneumonitis, myocarditis, or other immune-related adverse events that evolve unpredictably, and existing guidance emphasizes a low threshold for early recognition given the cumulative, variable-onset symptom nature [17]. However, this conservatism is not without cost: existing oncology telephone triage hotlines already direct a high proportion of calls to emergency departments, and systematic over-triage by an LLM-based adjunct could compound rather than relieve the emergency-department burden that nurse-led triage lines were designed to reduce [3]. Although all three LLMs correctly classified the Red cases in this study, no direct comparison with human triage performance was conducted. This finding should therefore be interpreted as concordance with the UKONS-based expert reference standard for the selected vignettes, rather than evidence of comparable or superior performance to human triage. Unlike the single-turn, text-based prompts used here, real-world human triage can incorporate tone of voice, clarifying questions, and direct assessment of patient-reported symptom severity. Notably, a recent study assessing ChatGPT’s ability to triage and respond to gynecologic oncology patient telephone calls showed over-triage in only 10% and under-triage in only 3.3% of the cases, with 100% accuracy in all 10 scenarios where the fellows recommended emergency department evaluation [18].

Strengths of this study include the use of a unanimous, three-expert gynecologic consultant consensus as the reference standard based on the established UKONS criteria, a vignette set deliberately spanning the major gynecologic malignancies and the principal categories of chemotherapy- and combined-therapy-related toxicity, and the parallel evaluation of three distinct, widely accessible LLM platforms under identical, non-iterative prompting conditions, which allowed the safety floor for high-acuity recognition to be assessed consistently across models. Several limitations nonetheless temper generalizability. The vignettes were fictitious, single-turn, and textually unambiguous, lacking the interactive questioning, incomplete symptom reporting, and tone-based cues present in genuine telephone encounters. Some presentations did not include all the information that may be required for a comprehensive UKONS-based triage assessment, such as symptom severity or grading, relevant blood counts, and other patient-specific clinical details where applicable. In real-world practice, such information may be obtained through structured questioning and review of clinical records and can influence the appropriate triage category. The absence of these details may therefore have affected the recommendations generated by the models and limits the extent to which the results reflect real-world telephone triage. An important limitation is the distribution of triage acuity levels within the vignette set. Of the twenty cases, twelve were classified as Red, five as Amber, and three as Green. Consequently, the dataset was weighted toward high-acuity presentations, while borderline and atypical cases, particularly those requiring differentiation between intermediate-urgency assessment and safe management at home, were underrepresented. This imbalance may have favored the identification of clear clinical emergencies and limits the conclusions that can be drawn regarding LLM performance in more nuanced triage decisions. Given that LLM performance may be sensitive to case complexity and the degree of clinical ambiguity, the results should be interpreted primarily as an initial assessment of emergency recognition rather than a comprehensive evaluation of telephone triage performance across all acuity levels. An additional limitation is that each clinical vignette was submitted to each LLM platform only once. Given the stochastic nature of LLM-generated outputs, repeated submissions of identical prompts may result in different clinical recommendations or triage classifications. Consequently, the present study evaluates the initial response generated by each platform rather than the consistency or reproducibility of its triage decisions. A further consideration concerns the use of free-tier, consumer-facing AI platforms. Although the model/version labels displayed by the interfaces were documented at the time of testing, the use of these platforms does not provide the same degree of control over model deployment as evaluation through fixed API model identifiers. Platform updates, automatic model-selection mechanisms, or fallback procedures may potentially affect the model serving individual requests. As provider-side routing information was not available, we cannot completely exclude this possibility. Accordingly, the results should be interpreted as an evaluation of the models as presented through their respective free-tier web interfaces on the testing date, rather than as a fully controlled comparison of fixed underlying model endpoints. An additional limitation concerns the composition of the expert reference panel. Although the UKONS tool was originally designed for nurse-led oncology telephone triage, the reference standard in this study was established exclusively by board-certified gynecologic consultants. While their specialist expertise provided a clinically informed assessment of treatment-related complications and the urgency of referral, experienced oncology triage nurses could have contributed an additional perspective reflecting the practical application of the UKONS framework in routine telephone triage. Consequently, the reference standard may not fully capture the decision-making processes and operational considerations of nurse-led triage services. Another methodological limitation is that the same three consultants established the UKONS-based reference standard and evaluated the LLM-generated responses. Although they were blinded to the platform that generated each response, this approach may have introduced incorporation bias, and the wording or style of the responses may have compromised effective blinding. Finally, the sample of 20 vignettes, while deliberately spanning major gynecologic malignancies and toxicity categories, is too small to support precise estimates of sensitivity, specificity, or inter-model comparative accuracy, and descriptive statistics alone were used accordingly.

These findings support consideration of LLMs as an adjunctive decision-support layer rather than an autonomous substitute for nurse-led triage. LLM-based tools that reliably escalate true emergencies while erring toward caution in ambiguous intermediate-risk scenarios could plausibly serve as pre-triage layers—prompting patients toward appropriate initial action and flagging calls for expedited nurse review—particularly for services without 24-h nurse coverage, consistent with national mandates for continuous specialist access during systemic anticancer therapy. Any such role, however, must remain supervised. The distinct toxicity profile of immune checkpoint inhibitors also adds complexity to any triage framework, human or artificial. Future work should therefore prioritize prospective validation in real patient populations, oncology-specific structured prompting or fine-tuning, and separate decision thresholds for patients on combined-therapy regimens versus chemotherapy alone before any wider clinical deployment is considered. Although all three LLMs correctly classified the twelve Red reference cases, this finding should be interpreted cautiously. The number of high-acuity scenarios was limited, and their relatively clear-cut clinical presentations may have contributed to a ceiling effect. Furthermore, the fictional and purposively constructed nature of the vignette set limits the generalizability of the observed results. Accordingly, the absence of misclassification among these cases should not be interpreted as evidence of reliable emergency recognition in routine clinical practice. Future studies should include a larger and more balanced set of vignettes, with deliberate enrichment of borderline, atypical, and clinically ambiguous presentations, to better assess the ability of LLMs to distinguish between different levels of urgency. Multiple independent repetitions of each clinical vignette using fixed, explicitly identified model versions and documented configurations need to be incorporated. Future studies should also incorporate experienced oncology triage nurses alongside physician specialists in the expert assessment panel, as well as compare LLM performance directly with real-world nurse-led triage decisions.

## 5. Conclusions

In conclusion, in this small study cohort, contemporary LLMs reliably flagged unambiguous emergent gynecologic oncology symptoms across both chemotherapy and combined-therapy contexts, preserving the safety floor of the UKONS triage framework, but remained relatively imprecise at the lower-acuity margins. This pattern supports a human-supervised, adjunctive role for LLMs within existing nurse-led, UKONS-based triage workflows rather than autonomous deployment, pending prospective validation in real-world patient populations.

## Figures and Tables

**Table 1:** Case vignettes—chemotherapy collective.

Cases	Description
1	Patient with ovarian cancer, second cycle of neoadjuvant chemotherapy with carboplatin/paclitaxel two days ago, calling the oncology triage line due to nausea and vomiting. The patient is able to eat/drink, but intake is significantly decreased. Antiemetics are taken as prescribed. Patient also reports two episodes of vomiting in 24 h. How do you consult the patient? Can this patient stay at home?
2	Patient with ovarian cancer, first cycle of adjuvant chemotherapy with carboplatin/paclitaxel 15 days ago, calling the oncology triage line due to epistaxis since yesterday. The patient is not on any anticoagulants, yet reports bruises on her extremities (with a negative trauma history). How do you consult the patient? Can this patient stay at home?
3	Patient with ovarian cancer, third cycle of neoadjuvant chemotherapy with carboplatin/paclitaxel 11 days ago, calling the oncology triage line due to fatigue, dysuria and body temperature of 38.6 °C. How do you consult the patient? Can this patient stay at home?
4	Patient with ovarian cancer, ninth cycle of chemotherapy with gemcitabine six days ago, calling the oncology triage line due to inability to drink and eat since yesterday due to mouth ulcers and pain swallowing. How do you consult the patient? Can this patient stay at home?
5	Patient with endometrial cancer, fourth cycle of chemotherapy with carboplatin/paclitaxel eight days ago, calling the oncology triage line due to generalized rash since yesterday that is constantly aggravating, in combination with severe pain and swelling. How do you consult the patient? Can this patient stay at home?
6	Patient with cervical cancer, second cycle of chemotherapy with carboplatin/paclitaxel three days ago, calling the oncology triage line due to a single incident of diarrhea today (first defecation of the day) with no further symptoms. No antidiarrheal medication has been taken so far. There has been no recent contact with anyone suffering from diarrhea. How do you consult the patient? Can this patient stay at home?
7	Patient with cervical cancer, eleventh cycle of chemotherapy with cisplatin/topotecan five days ago, calling the oncology triage line due to a persistent mild paresthesia and subjective weakness in the extremities (no loss of function, no pain). How do you consult the patient? Can this patient stay at home?
8	Patient with cervical cancer, third cycle of chemotherapy with etoposide/cisplatin two weeks ago, calling the oncology triage line due to a measured body temperature of 38.5 °C. The patient is feeling well and has no symptoms. How do you consult the patient? Can this patient stay at home?
9	Patient with uterine sarcoma, first cycle of chemotherapy with gemcitabine/docetaxel six days ago, calling the oncology triage line due to new-onset fatigue and restriction in strenuous activities. Fatigue is relieved by rest, though, and the patient is able to carry out work of light nature. How do you consult the patient? Can this patient stay at home?
10	Patient with cervical cancer, fourth cycle of chemotherapy with cisplatin/paclitaxel nine days ago, calling the oncology triage line due to no urine output since yesterday. How do you consult the patient? Can this patient stay at home?

**Table 2:** Case vignettes—combined therapy collective.

Cases	Description
1	Patient with ovarian cancer, third cycle of adjuvant therapy with carboplatin/paclitaxel/bevacizumab two weeks ago, calling the oncology triage line due to acute chest pain. How do you consult the patient? Can this patient stay at home?
2	Patient with ovarian cancer, fifth cycle of therapy with olaparib/bevacizumab, calling the oncology triage line due to constipation (no defecation) since yesterday. No other symptoms, no pain, no vomiting. How do you consult the patient? Can this patient stay at home?
3	Patient with ovarian cancer, second cycle of therapy with carboplatin/gemcitabine/bevacizumab five days ago, calling the oncology triage line due to new-onset dysuria and hematuria with incontinence and moderate reduction in output. How do you consult the patient? Can this patient stay at home?
4	Patient with ovarian cancer, eighth cycle of therapy with pembrolizumab/lenvatinib, calling the oncology triage line due to persistent dry cough for two months and shortness of breath with moderate exertion. How do you consult the patient? Can this patient stay at home?
5	Patient with endometrial cancer, third cycle of therapy with carboplatin/paclitaxel/dostarlimab four days ago, calling the oncology triage line due to acute visual disturbance since this morning. How do you consult the patient? Can this patient stay at home?
6	Patient with endometrial cancer, twelfth cycle of therapy with pembrolizumab nine days ago, calling the oncology triage line due to eleven episodes of acute bloody diarrhea since this morning. How do you consult the patient? Can this patient stay at home?
7	Patient with endometrial cancer, twentieth cycle of therapy with pembrolizumab/lenvatinib, calling the oncology triage line due to a single episode of vomiting yesterday and slight discomfort. The body temperature is 37.6 °C. How do you consult the patient? Can this patient stay at home?
8	Patient with cervical cancer, seventh cycle of therapy with pembrolizumab/bevacizumab 15 days ago, calling the oncology triage line due to increasing fatigue and inability to carry out any work activities. How do you consult the patient? Can this patient stay at home?
9	Patient with cervical cancer, third cycle of therapy with carboplatin/paclitaxel/bevacizumab one week ago, calling the oncology triage line due to new-onset shortness of breath with moderate exertion, in combination with mild disorientation and slight decrease in level of alertness. How do you consult the patient? Can this patient stay at home?
10	Patient with cervical cancer, last therapy with bevacizumab six months ago, now receiving no further therapy, calling the oncology triage line due to altered oral intake in association with weight loss (15 kg). How do you consult the patient? Can this patient stay at home?

**Table 3:** Telephone Triage Category Attribution—Chemotherapy Cases.

Cases	UKONS	OpenEvidence	ChatGPT	Google Gemini
1	1 Amber	Management at home with close follow-up within 24 h	Management at home, but only after a structured telephone assessment confirms clinical stability, adequate hydration, and the absence of red flags; telephone reassessment within a few hours or the same day after the intervention	Safe management at home, follow-up in 12–24 h to reassess response to adjusted antiemetic strategy
2	Red	Same-day, in-person evaluation	Urgent same-day evaluation	Immediate, same-day in-person evaluation
3	Red	Same-day, in-person evaluation	Same-day assessment	Immediate emergency evaluation
4	Red	Same-day, in-person evaluation	Same-day in-person assessment	Immediate direct evaluation
5	Red	Same-day, in-person evaluation	Urgent in-person assessment	Immediate, same-day in-person medical evaluation
6	1 Amber	Safe management at home; call back or seek urgent care in case of aggravation	Management at home, provided the patient is otherwise clinically well and has no red flags	Safe management at home with supportive care, provided the patient receives clear guidance on self-monitoring and red-flag symptoms
7	1 Amber	Remain at home with safety counseling, scheduled follow-up before the next cycle, clear instructions on when to call back or seek urgent care	May remain at home if the symptoms are genuinely mild, stable, symmetrical, with no objective neurological deficit or functional impairment and no red flags; prompt clinical review/laboratory assessment (ideally within 24 h)	May remain at home temporarily, schedule an outpatient visit within 24–48 h
8	Red	Same-day, in-person evaluation	Urgent same-day/in-person oncology assessment	Immediate, same-day in-person medical evaluation
9	Green	Outpatient reassurance, symptom screening, energy-conservation counseling, explicit return precautions for fever or other red-flag symptoms given the nadir timing	Can generally remain at home, provided there are no additional concerning symptoms and the patient is clinically stable	Safe management at home with supportive care, provided the patient receives clear guidance on self-monitoring and red-flag symptoms
10	Red	Same-day, in-person evaluation	Urgent presentation to the oncology emergency department	Immediate direct evaluation

All Green = self-care advice; 1 Amber = review within 24 h; 2 or more Amber = escalate to Red; Red = arrange for a face-to-face assessment as soon as possible.

**Table 4:** Telephone Triage Category Attribution—Combined Therapy Cases.

Cases	UKONS	OpenEvidence	ChatGPT	Google Gemini
1	Red	Urgent in-person evaluation	Acute medical assessment	Immediate in-person evaluation
2	Green	Safe management at home, re-evaluation in 24–48h	Safe management at home with supportive measures/laxative according to protocol and clear safety-netting, reassessment if there is no bowel movement over the next 24–48 h	Safely remain at home for initial conservative management, provided certain high-risk signs remain absent; check-in phone call in 24 h to reassess bowel activity and symptom status
3	Red	Urgent in-person evaluation	Urgent same-day medical assessment	Immediate direct clinical evaluation
4	1 Amber	Urgent office visit	Prompt in-person oncology/medical assessment, ideally the same day or within 24 h	Prompt in-person clinical evaluation
5	Red	Urgent in-person	Urgent same-day evaluation	Urgent, same-day face-to-face medical evaluation
6	Red	Urgent same-day evaluation	Urgent same-day/emergency assessment	Immediate emergency department evaluation
7	Green	Symptomatic home management, follow-up call in 24–48 h to confirm resolution	Home management is appropriate with clear safety-netting and reassessment if symptoms evolve	Urgent in-person evaluation
8	1 Amber	In-person evaluation	Same-day oncology evaluation	Immediate medical evaluation
9	2 Amber	Immediate in-person emergency evaluation	Urgent in-person assessment	Immediate emergency evaluation
10	Red	Same-day/next-day in-person evaluation	Prompt in-person oncology/medical assessment	Urgent direct clinical and diagnostic evaluation

All Green = self-care advice; 1 Amber = review within 24 h; 2 or more Amber = escalate to red; Red = arrange for a face-to-face assessment as soon as possible.

**Table 5:** 3 × 3 confusion matrix—OpenEvidence.

Reference/LLM	Red	Amber	Green
Red	12	0	0
Amber	2	1	2
Green	0	2	1

**Table 6:** 3 × 3 confusion matrix—ChatGPT.

Reference/LLM	Red	Amber	Green
Red	12	0	0
Amber	2	2	1
Green	0	1	2

**Table 7:** 3 × 3 confusion matrix—Google Gemini.

Reference/LLM	Red	Amber	Green
Red	12	0	0
Amber	2	2	1
Green	1	1	1

## Data Availability

The authors confirm that the key data supporting the findings of this study are available within the article and its accompanying tables. Due to the extensive length of the raw artificial intelligence generations, the complete, unedited prompts and full verbatim responses from each evaluated LLM are available from the corresponding author upon reasonable request.
